# Use of Antiviral Drugs to Reduce Household Transmission of Pandemic (H1N1) 2009, United Kingdom^1^

**DOI:** 10.3201/eid1706.101161

**Published:** 2011-06

**Authors:** Richard G. Pebody, Ross Harris, George Kafatos, Mary Chamberland, Colin Campbell, Jonathan S. Nguyen-Van-Tam, Estelle McLean, Nick Andrews, Peter J. White, Edward Wynne-Evans, Jon Green, Joanna Ellis, Tim Wreghitt, Sam Bracebridge, Chikwe Ihekweazu, Isabel Oliver, Gillian Smith, Colin Hawkins, Roland Salmon, Brian Smyth, Jim McMenamin, Maria Zambon, Nick Phin, John M. Watson

**Affiliations:** Author affiliations: Health Protection Agency, London, UK (R.G. Pebody, R. Harris, G. Kafatos, M. Chamberland, C. Campbell, J.S. Nguyen-Van-Tam, E. McLean, N. Andrews, P.J. White, E. Wynne-Evans, J. Green, J. Ellis, T. Wreghitt, S. Bracebridge, C. Ihekweazu, I. Oliver, G. Smith, C. Hawkins, M. Zambon, N. Phin, J.M. Watson);; Imperial College, London (P.J. White);; Public Health Wales, Cardiff, Wales, UK (R. Salmon);; Public Health Agency Northern Ireland, Belfast, Northern Ireland (B. Smyth);; Health Protection Scotland, Glasgow, Scotland (J. McMenamin)

**Keywords:** viruses, influenza, pandemic, prophylaxis, pandemic (H1N1) 2009, H1N1, household contacts, antimicrobial drugs, United Kingdom, research

## Abstract

The United Kingdom implemented a containment strategy for pandemic (H1N1) 2009 through administering antiviral agents (AVs) to patients and their close contacts. This observational household cohort study describes the effect of AVs on household transmission. We followed 285 confirmed primary cases in 259 households with 761 contacts. At 2 weeks, the confirmed secondary attack rate (SAR) was 8.1% (62/761) and significantly higher in persons <16 years of age than in those >50 years of age (18.9% vs. 1.2%, p<0.001). Early (<48 hours) treatment of primary case-patients reduced SAR (4.5% vs. 10.6%, p = 0.003). The SAR in child contacts was 33.3% (10/30) when the primary contact was a woman and 2.9% (1/34) when the primary contact was a man (p = 0.010). Of 53 confirmed secondary case-patients, 45 had not received AV prophylaxis. The effectiveness of AV prophylaxis in preventing infection was 92%.

## Abstract 2

TOC Summary: Early treatment of primary case-patients and prophylaxis of household contacts provides effective protection.

Following emergence of pandemic influenza A (H1N1) 2009 in North America in spring 2009 (1,2), global spread of the virus was rapid (3,4). In the United Kingdom, the first confirmed cases were detected in travelers returning from Mexico (5). The United Kingdom implemented a containment strategy until July 2009 that involved rapid case ascertainment, early treatment with antiviral drugs (AVs), and postexposure prophylaxis of patients’ close contacts.

One key uncertainty was the transmissibility of the virus in household settings. Household-based studies of avian influenza previously provided a measure of transmissibility of newly emerging influenza viruses and also of the effectiveness of AVs in reducing spread (6). Early reports on pandemic (H1N1) 2009 have provided information on household transmission (7–11). Although most are from settings where AVs were not used (8,10) or where only a limited number of households were recruited (7,9), early work suggests that AVs had some effect on spread (11,12).

A detailed investigation of the first few 100 (FF100) case-patients and their close contacts (13) was undertaken across the United Kingdom beginning in April 2009 to gain an early understanding of the clinical and epidemiologic parameters of pandemic (H1N1) 2009 (14). Following the publication of early FF100 findings (5,11,15), we report the final results from ≈300 UK households of key household transmission characteristics.

## Methods

The FF100 study has been described in detail (15,16). This study was a prospective investigation of the first laboratory-confirmed cases and patients’ household contacts to determine key parameters such as virologic and clinical secondary attack rates (SARs) and effectiveness of AVs.

### Definitions

Three case definitions were used: 1) virologically confirmed cases were persons testing positive for pandemic (H1N1) 2009 virus by specific reverse transcription PCR (RT-PCR) on respiratory swab; 2) influenza-like illness (ILI) cases were persons experiencing history of fever and >1 respiratory symptom (dry cough, productive cough, coryza, shortness of breath, or sneezing) within 2 weeks of onset of the confirmed household primary case; and 3) acute respiratory infection (ARI) cases were persons experiencing >1 respiratory symptom (as defined above) and/or fever within 2 weeks of onset of the confirmed household primary case. A household contact was any person who lived in the same household as a confirmed primary case-patient and >1 overnight stay after onset of illness in the person who was the primary case-patient (16).

A household was defined as the primary case-patient plus all household contacts. For a household, a virologically confirmed primary case was the case-patient with first date of onset within that household. A secondary case was any case-patient with date of onset >24 hours after date of onset of primary case. If a patient’s onset of illness was <24 hours of onset of the primary case, it was classified as co-primary. A similar approach was followed for clinically confirmed secondary cases, with clinical co-primary cases excluded.

Secondary cases were defined as case-patients who had received prophylaxis if AVs were administered <24 hours before illness onset. Any asymptomatic contact who received AVs was classified as having prophylaxis. For a small number of contacts with non–case-defining symptoms before starting AVs, it was not possible to distinguish prophylaxis and treatment. These contacts were excluded for AV analyses.

### Case Ascertainment

Initially, all patients with virologically confirmed cases detected in the United Kingdom were included in the FF100 dataset, and their households were followed up. As case numbers grew rapidly, convenience sampling was undertaken before closure of FF100 on June 21, 2009.

### Collection of Epidemiologic Information

Information on case-patients was collected at 2 time points. Initial information was collected as soon as possible after a positive laboratory result was reported. Data were collected directly from case-patients or their parent or guardian by public health workers in person or by telephone interview. Information collected included demographics, clinical history (date of illness onset, signs and symptoms), medical history (including 2008–09 seasonal trivalent influenza vaccine or AV use), and underlying medical conditions. Inactivated trivalent influenza vaccines from various manufacturers are used in the United Kingdom with composition determined by World Health Organization recommendations.

Case-patients provided details of close household contacts. At initial interview, contacts were asked about their contact history with the primary case-patient; clinical history, including recent respiratory symptoms with dates of onset and treatment; medical history, including underlying medical conditions; and use of AVs with dates of administration.

Daily telephone follow-up of contacts was undertaken for 7 days. If any respiratory symptoms developed, contacts were instructed to speak to their general practitioners for prompt investigation, including collection of respiratory swab specimens. Swab samples were also inadvertently obtained from several contacts who did not have case-defining illness. To ensure that all contacts testing positive for pandemic (H1N1) 2009 virus were identified, the FF100 database and Health Protection Agency (HPA) laboratory reports of confirmed cases were compared.

Final follow-up of case-patients and household contacts was undertaken >2 weeks after to gather information on possible complications, final outcome (e.g., illness, death, and recovery), and use of AVs and antimicrobial drugs. For scheduled telephone follow-up, calls were attempted for a minimum of 3 consecutive days before the patient was classified as lost to follow-up. Information was gathered on a hard-copy questionnaire or entered directly into a Web-enabled database. Data verification and quality assurance were undertaken through standard data entry checks, double entry, and internal and external consistency checks.

### Statistical Analysis

Single-person households were excluded from household analysis. SAR was calculated for clinical illness (ILI and ARI) and confirmed infection. The cumulative household SAR was defined as the total number of secondary cases in a household divided by number of household members at risk (excluding primary and co-primary cases) 14 days after onset in the primary case-patient. Household SAR was calculated by age group (<16 years, 16–49 years [reference group], >50 years), gender, AV prophylaxis (yes or no), and timing of treatment for the primary case-patient (<48 hours vs. >48 hours) through univariate logistic regression analyses for the different endpoints. Multivariate analyses were also performed, adjusted for the aforementioned variables, and model fit assessed by using the Hosmer-Lemeshow goodness-of-fit test. Because confirmed SAR may be affected by failure to obtain swabs from symptomatic contacts, observed positivity rates in the ARI and nonsymptomatic groups were used to adjust for this possibility.

A survival analysis was undertaken to determine the effect of prophylaxis on household SAR while accounting for timing of administration. A contact enters the model with time zero at index onset, and survival time is defined up until onset of disease in the contact (failure), or excluded at the end of the 2-week follow-up period. AV prophylaxis exposure was treated as a time-varying covariate, and for each contact, survival time was split into pre-AV and AV prophylaxis periods. The hazard ratio of becoming a secondary case-patient when AV prophylaxis was given was estimated by using Cox regression, adjusted for age, sex, and AV treatment of the primary case-patient <48 hours. This approach accounted for prophylaxis not usually being given to contacts until the case-patient was identified by health services.

### Laboratory Confirmation

Respiratory samples from influenza patients were analyzed for pandemic influenza A (H1N1) 2009 and seasonal influenza viruses by RT-PCR. Combined nose and throat swab specimens were collected from patients who had signs and symptoms of suspected infection. These specimens were sent to a designated UK laboratory performing real-time RT-PCR for pandemic (H1N1) 2009 virus. Pandemic (H1N1) 2009 diagnosis was confirmed before June 2009 by sequencing the influenza A PCR amplicon (17), and from June onwards by real-time PCR of a swine lineage N1 (18).

### Ethical Considerations

This observational study was undertaken as part of management of a national outbreak. The work was done under National Health Service Act 2006 (section 251), which provides statutory support for disclosure of such data by NHS and data processing data by HPA for communicable disease control. Health Protection Scotland remains embedded as part of NHS, and outbreak and investigation data were shared as part of the coordination of national outbreaks.

## Results

### Recruitment and Follow-up of Households

**Figure 1:**
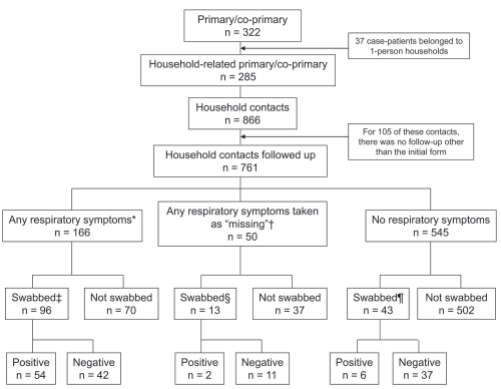
Flowchart of pandemic (H1N1) 2009 case-patients and household contacts, including contacts with respiratory symptoms, contacts from whom swab specimens were collected, and PCR result, United Kingdom, 2009. *Symptom onset date <2 weeks after index case-patient symptom onset; †46 persons had symptom onset date >2 weeks after index case-patient and 4 had missing symptom onset date; ‡5 persons had swabs taken >2 weeks after index case-patient symptom onset, and 3 had positive test results; §2 persons (neither positive) had swabs taken >2 weeks after index case-patient symptom onset; ¶3 persons (none positive) had swabs taken >2 weeks after index case-patient symptom onset.

A total of 322 confirmed primary and co-primary case-patients were identified in 296 households (Figure 1). Of these 296 households, 37 were single-person. Case-patients from single-person households were older (mean age 27.4 vs. 19.7 years in other households; p = 0.003) with a nonsignificant trend toward males (64.9% vs. 50.2%; p = 0.092). Single-person households were excluded from further analysis, leaving 259 primary and 26 co-primary case-patients in 259 households (Figure 1).

The total number of household contacts identified was 866. Of these, 105 (12.1%) declined to participate or were lost to follow-up (Figure 1), with no significant differences in age (p = 0.32) and sex (p = 0.47) between those followed and not followed-up. Distribution of household sizes, primary cases, contacts, and secondary cases is shown in Table 1.

**Table 1:** Household size of case-patients with pandemic (H1N1) 2009 virus infection, United Kingdom, 2009

No. persons in household	No. households	No. primary and co-primary case patients	No. contacts	No. secondary case-patients
2	42	44	40	2
3	46	51	87	6
4	76	81	223	15
5	28	31	109	7
6	18	20	88	2
7	12	17	67	13
8	7	10	46	5
9	4	4	32	1
10	4	5	35	6
11	2	2	20	2
15	1	1	14	3
Total	240	266	761	62

### Household and Primary Case-Patient Characteristics

Average household size was 4 people (SD = 2.1), with a median size of 4 (interquartile range [IQR] 3–5) (Table 1). A comparison of age, gender, and AV use of primary case-patients, co-primary case-patients, and contacts is provided in Table 2.

**Table 2:** Primary and co-primary confirmed case-patients with pandemic (H1N1) 2009 virus infection and household contacts, by sex, age, and prophylaxis status, United Kingdom, 2009*

Variable	No. (%) primary and co-primary case-patients	No. (%) contacts
Sex, n = 1,030		
M	143 (50.2)	364 (48.9)
F	142 (49.8)	381 (51.1)
Age, y		
<16	154 (54.0)	212 (27.9%)
16–49	114 (40.0)	378 (49.7)
>50	17 (6.0)	171 (22.5)
Prophylaxis, n = 843		
No	253 (98.8)	132 (22.5)
Yes	3 (1.2)	455 (77.5)
Total	285	761

*n = 1,046 except as indicated.

Of the primary case-patients, 245 (95.7%) had received AV treatment (of whom 116/118 with information had received oseltamivir). Among treated case-patients, 104 (42.4%) had started treatment <48 hours of disease onset, with median time to AV treatment of 3 days (IQR 1–5).

### Household Close Contacts

**Figure 2:**
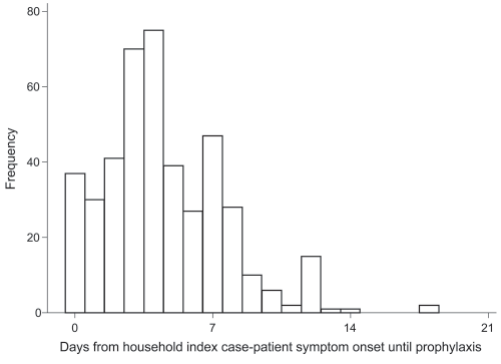
Days from symptom onset date of household primary case-patient with pandemic (H1N1) 2009 virus infection until antiviral prophylaxis started, N = 352, United Kingdom, 2009.

The age and gender distribution of the 761 followed-up household contacts are summarized in Table 2. Information on AV prophylaxis was available for 587 contacts (Table 2, Table 3); of the 444 contacts who named the AV they received, 435 received oseltamivir. Mean number of days from onset in the primary case-patient to starting prophylaxis in contacts was 4.4 days (SD 4.9, median 4 days, IQR 2–6 days) (Figure 2). Compliance for use of AVs found 255 contacts with information on prophylaxis start and end dates, with a median time to receiving AV of 9 days (IQR 8–10). Only 8 contacts received treatment for <5 days.

**Table 3:** Confirmed SAR of pandemic (H1N1) 2009 virus infection, according to time antiviral drug prophylaxis began after onset of illness in primary case-patient, plus timing of secondary cases after onset of primary case, United Kingdom, 2009*

Timing	No. contacts	No. secondary case-patients at 14 d	SAR, % (95% CI)	No. (%) secondary case-patients			
2 d	3–4 d	5–7 d	>7 d
No prophylaxis	143	45	31.5 (24.0–39.8)	15	12	10	8
Day 0	57	1	1.8 (0.0–9.4)	0	0	1	0
Days 1–2 (<48 h)	81	4	4.9 (1.4–12.2)	0	3	1	0
Day 3–7 (inclusive)	214	3	1.4 (0.3–4.0)	NA	0	3	0
>7 d	92	0	0.0 (0.0-3.9)	NA	NA	NA	0
Total case-patients	587	53	9.0 (6.8–11.7)	15 (2.6)	15 (2.6)	15 (2.6)	8 (1.4)

*SAR, secondary attack rate; CI, confidence interval; NA, not applicable.

### Household Secondary Attack Rates

Household contacts in whom respiratory symptoms developed within 2 weeks and from whom swab samples were collected are summarized in Figure 1. Overall, of 761 household contacts, 166 had ARI symptoms, 62 of whom were confirmed secondary case-patients, with a SAR of 8.1% (Table 4). Among those without ARI, 43 provided swab samples, 6 of whom had positive test results. The positivity rate in those with and without ARI that were tested was projected onto non-swabbed ARI patients to give an adjusted confirmed SAR of 13.8%. The SAR, adjusted for age and sex, was 16.7%.

**Table 4:** Univariate and multivariate analysis of pandemic (H1N1) 2009 virus infection SAR for virologically confirmed cases of pandemic (H1N1) 2009 virus infection, by gender, age group, and prophylaxis, United Kingdom, 2009*

Variable	No. contacts†	No. secondary case-patients	Univariate analysis			Multivariate analysis	
			SAR, % (95% CI)	p value‡		OR (95% CI)	p value
Sex, n = 745							
M	364	37	10.2 (7.5–13.7)			1.0, baseline	
F	381	25	6.6 (4.0–10.7)	0.08		1.0 (0.5–2.0)	0.96
Age, y							
<16	212	40	18.9 (14.2–24.7)			18.2 (3.9–85.5)	
16–49	378	20	5.3 (3.1–9.0)			3.5 (0.7–16.2)	
>50	171	2	1.2 (0.3–4.7)	<0.001		1.0, baseline	<0.001
Prophylaxis, n = 587							
No	143	45	31.5 (24.4–39.5)			1.0, baseline	
Yes	444	8	1.8 (0.8–3.9)	<0.001		0.05 (0.02–0.09)	<0.001
Primary case-patient treatment							
>48 h	453	48	10.6 (8.1–13.8)			1.0, baseline	
<48 h	308	14	4.5 (2.5–8.1)	0.003		0.30 (0.13–0.68)	0.004
Total	761	62	8.1 (6.4–10.3)				

*n = 761 except as indicated. Hosmer-Lemeshow goodness-of-fit test for multivariate model, p = 0.751. SAR, secondary attack rate; CI, confidence interval; OR, odds ratio. †Excludes co-primary cases. ‡Indicates overall p value for differences by group.

Univariate analysis revealed a significantly higher confirmed SAR for patients aged <16 years and for those 16–49 years, compared to those >50 years. The SAR in male patients was higher than female patients, but the difference was not significant (Table 4). Most secondary case-patients (86.8%, 45/53) had not received prophylaxis; contacts who had not received AV prophylaxis had a significantly higher confirmed SAR than those who had (Table 4). Contacts who received prophylaxis <2 days after onset in the primary case-patient had a nonsignificantly higher SAR than those who received therapy later (Table 3), although the study did not have sufficient statistical power to detect such differences. The confirmed SAR was significantly lower in contacts whose primary case-patient had received treatment <48 hours of onset rather than after 48 hours (Table 4).

The confirmed SAR by age of primary case-patients is shown in Table 5. Confirmed SAR was high among those <16 years of age, whether the primary case-patient was a child or an adult. Similarly, SAR was low among adults, whether the primary case-patient was a child or an adult (Table 5). When transmission from adults to children was analyzed by gender, a significant difference was found for SARs in children according to sex of the adult primary case-patient: 33.3% (10/30, 95% confidence interval [CI] 17.3–52.8) for female primary case-patients and 2.9% (1/34, 95% CI 0.1–15.3) for men (odds ratio 16.5, 95% CI 2.0–138.8; p = 0.010).

**Table 5:** Pandemic (H1N1) 2009 virus infection SAR, by age of patient with virologically confirmed primary case, United Kingdom, 2009*

Transmission†	No. contacts	No. secondary case-patients	SAR, % (95% CI)
Child to child	148	29	19.6 (13.5–26.9)
Child to adult	318	9	2.8 (1.3–5.3)
Adult to adult	231	13	5.6 (3.0–9.4)
Adult to child	64	11	17.2 (8.9–­28.7)

*SAR, secondary attack rate; CI, confidence interval. †Primary case-patient to contact.

Multivariate analysis shows the adjusted odds for a virologically confirmed secondary case were significantly higher for children <16 years of age than for adults. In addition, contacts who received AV prophylaxis had a significantly reduced risk of confirmed infection than those not treated (Table 4). Finally, the adjusted odds of a secondary case-patient were significantly lower when the primary case-patient had received treatment <48 hours of onset.

### SAR for Clinically Confirmed Cases of ILI and ARI

For the ILI outcome, 259 households yielded an additional 16 cases defined as co-primaries. Seventy-eight clinically confirmed secondary cases occurred among 745 contacts for an overall household ILI SAR of 10.5% (Table 6).

**Table 6:** Univariate and multivariate analysis of pandemic (H1N1) 2009 virus infection SAR for clinically confirmed cases of influenza-like illness, by gender, age group, and prophylaxis, United Kingdom, 2009*

Variable	No. contacts†	No. secondary case-patients	Univariate analysis			Multivariate analysis	
			SAR % ( 95% CI)	p value‡		OR (95% CI)	p value
Sex, n = 730							
M	357	33	9.2 (6.7–12.7)			1.00, baseline	
F	373	45	12.1 (7.9–18.1)	0.22		2.6 (1.4–4.9)	0.003
Age group, y							
<16	204	38	18.6 (13.9–24.6)			7.8 (2.7–22.1)	
16–49	371	32	8.6 (5.4–13.5)			2.7 (1.0–7.4)	
>50	170	8	4.7 (2.2–9.8)	<0.001		1.00, baseline	<0.001
Prophylaxis, n = 573							
No	129	56	43.4 (35.1–52.1)			1.0, baseline	
Yes	444	18	4.1 (2.3–7.1)	<0.001		0.05 (0.02–0.09)	<0.001
Primary case-patient treatment							
>48 h	445	55	12.4 (9.6–15.8)			1.0, baseline	
<48 h	300	23	7.7 (4.7–12.2)	0.040		0.78 (0.42–1.48)	0.458
Total	745	78	10.5 (8.5–12.9)				

*n = 745 except as indicated. Hosmer-Lemeshow goodness-of-fit test for multivariable model, p = 0.291. SAR, secondary attack rate; CI, confidence interval; OR, odds ratio. †Excludes co-primary case-patients. ‡Indicates overall p value for differences by group.

For the ARI outcome, a further 26 ARI cases were defined as co-primaries. In the 259 households, of 719 contacts, 120 secondary case-patients resulted for an ARI SAR of 16.7% (Table 7). The effect of age, AV prophylaxis of contacts, and early treatment of case-patients were generally similar for both ILI and ARI clinical endpoints compared to virologically confirmed endpoints in both univariate and adjusted analysis (Table 6, Table 7).

**Table 7:** Univariate and multivariate analysis of pandemic (H1N1) 2009 SAR infection for acute respiratory infection, by gender, age group and prophylaxis, United Kingdom, 2009*

Variable	No. contacts†	No. secondary case-patients	Univariate analysis			Multivariate analysis	
SAR, % (95% CI)	p value‡	OR (95% CI)	p value
Sex, n = 704							
M	339	56	16.5 (12.9–20.9)			1.0, baseline	
F	365	64	17.5 (12.5–24)	0.72		1.9 (1.0–3.5)	0.04
Age, y							
<16	194	49	25.3 (15.4–38.6)			7.0 (3.0–21.0)	
16–49	359	56	15.6 (9.2–25.2)			3.6 (1.5–8.8)	
>50	166	15	9.0 (5.5–14.4)	<0.001		1.0, baseline	0.001
Prophylaxis, n = 549							
No	106	80	75.5 (66.4–82.7)			1, baseline	
Yes	443	34	7.7 (4.5–12.7)	<0.001		0.02 (0.01–0.03)	<0.001
Primary case-patient treatment							
>48 h	435	79	18.2 (14.8–22.1)			1, baseline	
<48 h	284	41	14.4 (10.1–20.3)	0.019		1.7 (0.9–3.1)	0.11
Total	719	120	16.7 (14.1–19.6)				

*n = 719 except as indicated. Hosmer-Lemeshow goodness-of-fit test for multivariate model, p = 0.392. SAR, secondary attack rate; CI, confidence interval; OR, odds ratio. †Excludes coprimary case-patients. ‡Indicates overall p-value for differences by group.

### Survival Analysis of Prophylaxis

**Figure 3:**
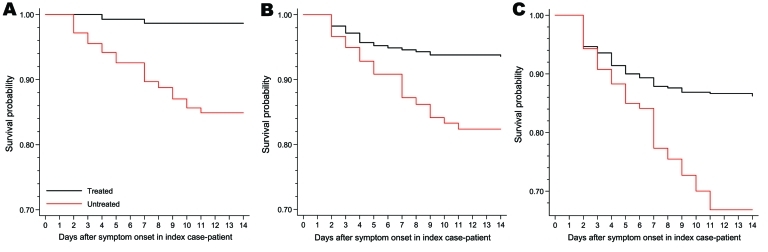
Kaplan-Meier graphs of days from symptom onset in index case-patient until onset of symptoms in secondary case-patients, United Kingdom, 2009. A) Virologically confirmed pandemic (H1N1) 2009; B) clinical influenza-like illness; C) acute respiratory infection.

The hazard ratio (HR) of becoming a confirmed secondary case-patient when receiving AV drugs was 0.08 (95% CI 0.02–0.27). Results were similar after adjusting for AV treatment of the primary case-patient, age, and sex (HR 0.09, 95% CI 0.03–0.32). When looking at ILI endpoint, the unadjusted HR was 0.27 (95% CI 0.13–0.56) and adjusted HR was 0.27 (95% CI 0.13–0.57) and for ARI, the unadjusted HR was 0.31 (95% CI 0.18–0.52) and adjusted was 0.27 (95% CI 0.15–0.48). The Kaplan-Meier plots for the 3 endpoints are shown in Figure 3 and multivariate survival analysis results in Table 8.

**Table 8:** Multivariable survival analysis of for pandemic (H1N1) 2009 virus infection SAR with virologic, influenza-like-illness, and acute respiratory infection endpoints, by gender, age group, and prophylaxis, United Kingdom, 2009*

Variable	Hazard ratio (95% confidence interval)		
	Virologic	Influenza-like illness	Acute respiratory infection
Sex			
M	1 (reference)	1 (reference)	1 (reference)
F	0.97 (0.56–1.69)	1.77 (1.08–2.89)	1.30 (0.90–1.90)
Age, y			
<16	4.23 (2.35–7.62)	2.78 (1.68–4.61)	1.90 (1.29–2.81)
16–49	1 (reference)	1 (reference)	1 (reference)
>50	0.33 (0.08–1.42)	0.47 (0.18–1.22)	0.54 (0.29–1.00)
Antiviral drug prophylaxis			
Untreated	1 (reference)	1 (reference)	1 (reference)
Treated	0.09 (0.03–0.32)	0.27 (0.13–0.57)	0.27 (0.15– 0.48)
Index case-patient treatment			
>48 h	1 (reference)	1 (reference)	1 (reference)
<48 h	0.45 (0.23–0.87)	0.72 (0.42–1.23)	0.99 (0.66–1.50)

*SAR, secondary attack rate.

In most households, either all members received prophylaxis (122/206, 59.2%) or none at all (30/206, 14.6%). In discordant households, where some received prophylaxis and some did not (54/206, 26.2%), virologically confirmed SAR was similar to the main analysis: the SAR was 41.7% (95% CI 30.8%–53.4%) in those not receiving and 3.2% (95% CI 0.9%–7.9%) in those receiving prophylaxis. Survival analyses were repeated to allow for clustering within households, with the CIs being marginally wider.

## Discussion

This study involved the prospective follow-up of households during the UK containment phase for pandemic (H1N1) 2009. We found a moderately high, virologically confirmed SAR with higher clinical (ILI and ARI) endpoints. Age-specific differences for SARs were significant; the SAR was highest among children. The SARs for child contacts were higher when adult women were the primary case-patients than when men were. Finally, most secondary case-patients had not received AV prophylaxis, and AV administration to household contacts substantially reduced the risk for infection.

This study found an overall virologically confirmed household SAR of 8%, similar to results for an earlier study involving the FF100 (11): SAR reached 34% among contacts who did not receive AV prophylaxis. The SAR increased further for clinical endpoints. These SARs for those who did not receive AVs compare to results of a study in Kenya which reported a confirmed household SAR of 26% (10) in a population without widespread use of AV prophylaxis. Another study in Japan (7), where >90% of contacts had received AV prophylaxis, reported a virologically confirmed SAR of only 5%. Other studies have used clinical endpoints, such as in the United States (8), where a clinical SAR of 10% was reported after 7 days. These findings compare to household SARs found for seasonal influenza in historical studies, ranging from 18% (19) to 22% (20). Although these studies had similar design, there are several possible explanations for our results, such as differences in case definition, a different period of follow-up, differences in ascertainment of secondary cases, and differences in AV use. Our observed SAR among those who did not receive prophylaxis is higher than that previously observed for seasonal influenza and suggests a substantial proportion of close contacts were infected with pandemic (H1N1) 2009 virus. Serologic studies will provide important insights into the rates of infection (both symptomatic and asymptomatic in a household setting).

Recent publications have explored the possibility of using household data to estimate AV effectiveness for seasonal influenza (21,22). Our study provides evidence that AV prophylaxis of household contacts significantly reduces SAR for all endpoints, updating earlier work (11). Most secondary cases occurred in contacts who had not yet received AV prophylaxis after onset of illness in the primary case-patient, with a very high SAR observed in those that had not received AV for all endpoints, due to the delay for many before prophylaxis was started. The adjusted survival analysis took into account the confounding effect of time to prophylaxis and demonstrated that AVs are effective for all endpoints. Other studies in Japan (7), the United States (9,12), Hong Kong, China (23), and Germany (24) have attempted to determine the effectiveness of postexposure prophylaxis for pandemic influenza. Most show a statistically nonsignificant positive effect of AVs (7,9). Studies concerning AV effectiveness for seasonal influenza, in particular a large placebo-controlled household study, found that postexposure prophylaxis reduced the incidence of infection in close household contacts by 89% (25). Our study demonstrates that timely administration of AVs to close contacts provides significant protection against clinical disease.

Our study found clear age-specific differences in SAR, with a much higher household SAR in children than in the elderly. This age-specific pattern is also replicated, at least partially, by seasonal influenza: Longini reported a SAR of 24% in those <18 years of age and a rate of 14% in those >18 years (26). The high household SARs in children in the present study, illustrates the susceptibility of this subgroup and is consistent with general practice consultation data, laboratory surveillance data, and results of school outbreak investigations (27,28). The observation of very low SAR in those >50 years, who have also had household exposure to a confirmed case, demonstrates protection afforded by cross reacting H1N1 influenza antibodies from prior exposure to H1N1 subtypes circulating in the period before 1957 (29,30).

This study found that SAR was significantly lower when the primary case-patient had received rapid AV treatment, before and after adjustment for prophylaxis of contacts. The observation is biologically plausible as studies demonstrate early AV use reduces virus shedding (31). This may translate into reduced likelihood of secondary transmission and supports rapid treatment of patients to reduce household transmission. The observation that SARs from child to child and from adult to child (>20%) were similar, yet at least 4-fold higher than from child to adult or adult to adult, is also consistent with the increasing prevalence of cross-reacting antibodies against pandemic (H1N1) 2009 virus with age (32). Children are known to excrete influenza virus in higher titers and for a longer period than adults (33,34), and social play between children often entails very close contact, so an SAR of 21% from child-to-child is expected. The SAR, however, for adult-to-child transmission was just as high, particularly among female primary case-patients, which suggests that despite lower virus titers and shorter duration of excretion, women transmitted pandemic (H1N1) 2009 infection as efficiently as child primary case-patients. This suggests adult respiratory hygiene is suboptimal in the home environment.

This study has several strengths: this is one of the largest pandemic influenza household studies published to date, and active follow-up was undertaken with daily telephone calls to ensure timely clinical investigation with swab collection to maximize case ascertainment. There are, however, limitations. First, not all those who had respiratory symptoms develop had throat swabs done, leading to under-ascertainment of confirmed secondary case-patients. Adjustments have been made to account for this. Second, case finding was based on a screening algorithm requiring fever. Thus, primary cases of pandemic influenza without fever would have been excluded; however, all clinical endpoints were gathered from secondary case-patients. Third, this article presents information only on clinical and virologic endpoints. There is now evidence that a substantial proportion of persons exposed to a primary case-patient will have asymptomatic or very mildly symptomatic infection. This requires serologic investigation (30). Fourth, because data were captured as part of the acute public health response, data gathering was undertaken through multiple interviewers. Missing data were minimized by final follow-up of case-patients and contacts, and the demographic profile was not indicative of a systematic bias that might invalidate the results. Fifth, if a primary case-patient was confirmed quickly, their contacts may have avoided further contact, whereas if the primary case-patient was identified later, close contact may not have been avoided. However, a time-varying survival analysis found no significant difference for contacts not receiving AV. Sixth, information concerning prior respiratory disease in contacts was not gathered, and some persons may have had prior exposure to pandemic (H1N1) 2009. However, this is unlikely because pandemic transmission was not yet widespread when our data were collected, and this should not have been a major potential confounding factor. Finally, we assumed household secondary case-patients acquired their infection after contact with a defined primary case-patient in the household, rather than in the community. Although more advanced statistical methods do exist to take into account these competing transmission risks (26,35), this study was undertaken at a stage when community transmission was limited so this contribution is assumed to be minimal.

In conclusion, we demonstrate transmission of pandemic influenza in the household setting in the United Kingdom during the containment phase. Household SARs were generally higher than those of seasonal influenza. Timely AV treatment of primary case-patients and prophylaxis was effective in protecting household contacts, although delayed administration of AV did allow spread. Prompt AV administration (either as treatment or prophylaxis) reduces symptomatic SARs.
